# Quantitative Ultrasound Characterization of Tumor Cell Death: Ultrasound-Stimulated Microbubbles for Radiation Enhancement

**DOI:** 10.1371/journal.pone.0102343

**Published:** 2014-07-22

**Authors:** Hyunjung Christina Kim, Azza Al-Mahrouki, Alborz Gorjizadeh, Ali Sadeghi-Naini, Raffi Karshafian, Gregory J. Czarnota

**Affiliations:** 1 Department of Physical Sciences, Sunnybrook Health Sciences Centre, Toronto, Ontario, Canada; 2 Department of Radiation Oncology, Odette Cancer Centre, Sunnybrook Health Sciences Centre, Toronto, Ontario, Canada; 3 Department of Medical Biophysics, Faculty of Medicine, University of Toronto, Sunnybrook Health Sciences Centre, Toronto, Ontario, Canada; 4 Department of Radiation Oncology, Faculty of Medicine, University of Toronto, Toronto, Ontario, Canada; 5 Department of Physics, Ryerson University, Toronto, Ontario, Canada; NIH, United States of America

## Abstract

The aim of this study was to assess the efficacy of quantitative ultrasound imaging in characterizing cancer cell death caused by enhanced radiation treatments. This investigation focused on developing this ultrasound modality as an imaging-based non-invasive method that can be used to monitor therapeutic ultrasound and radiation effects. High-frequency (25 MHz) ultrasound was used to image tumor responses caused by ultrasound-stimulated microbubbles in combination with radiation. Human prostate xenografts grown in severe combined immunodeficiency (SCID) mice were treated using 8, 80, or 1000 µL/kg of microbubbles stimulated with ultrasound at 250, 570, or 750 kPa, and exposed to 0, 2, or 8 Gy of radiation. Tumors were imaged prior to treatment and 24 hours after treatment. Spectral analysis of images acquired from treated tumors revealed overall increases in ultrasound backscatter intensity and the spectral intercept parameter. The increase in backscatter intensity compared to the control ranged from 1.9±1.6 dB for the clinical imaging dose of microbubbles (8 µL/kg, 250 kPa, 2 Gy) to 7.0±4.1 dB for the most extreme treatment condition (1000 µL/kg, 750 kPa, 8 Gy). In parallel, *in situ* end-labelling (ISEL) staining, ceramide, and cyclophilin A staining demonstrated increases in cell death due to DNA fragmentation, ceramide-mediated apoptosis, and release of cyclophilin A as a result of cell membrane permeabilization, respectively. Quantitative ultrasound results indicated changes that paralleled increases in cell death observed from histology analyses supporting its use for non-invasive monitoring of cancer treatment outcomes.

## Introduction

The ability to monitor the early response of a cancer to treatment is crucial in determining whether adjustment to a treatment plan is necessary. The most common method of examining treatment response is a biopsy which assesses the cellular characteristics of tumor [1]–[3]. However, the inherent cost of this highly precise response validation tool is that it is an invasive and time-consuming procedure not accepted by patients for response monitoring which makes it often impractical for use. In addition, biopsy often requires imaging guidance using computed tomography, magnetic resonance, or ultrasound guidance for improved accuracy [4]–[7]. An alternative to biopsy that has been investigated, which has the potential to overcome current challenges of tissue monitoring, is the use of quantitative ultrasound. Quantitative ultrasound is a non-invasive method, which can provide rapid therapy response assessment [8]–[9]. It has a distinct advantage in that it can be used to evaluate early treatment responses and provide medical practitioners with a suitable future direction with regards to considering salvage therapy or changes in the primary treatment thereby potentially improving patient prognosis [10]–[13]. Previous studies have demonstrated that quantitative analysis of ultrasound data can be used to characterize microscopic scatterers of ultrasound based on signal intensity, scatterer size, and scatterer concentration, which can sufficiently distinguish damaged cells from healthy cells [11], [14]–[17]. This has been demonstrated recently *in vivo* using conventional diagnostic frequencies (7–10 MHz) where apoptotic cell death was successfully distinguished when using radiation therapy and chemotherapy, and previously with photodynamic therapy [11], [15]. There are also other non-invasive imaging modalities used for detecting the results of treatment-related microscopic cellular changes, such as magnetic resonance imaging, positron emission tomography, and optical imaging [11], [18]–[20]. However, the low cost, portability, lack of need of contrast agents, and rapid image acquisition and processing makes quantitative ultrasound imaging appealing amongst these other methods for the detection of cell death [8]–[9].

Apoptosis, or programmed cell death, is a process characterized by distinct structural changes at various stages [21]. In the earliest stage, compaction and segregation of nuclear material (i.e. chromatin) occur, followed by fragmentation of the nucleus and budding of membrane-bound apoptotic bodies at the late stages [21]. These changes in morphology generate scattering sources that can reflect ultrasound differently from viable cells [21]. Several studies have confirmed that normalized radiofrequency power spectra from ultrasound can be quantitatively analyzed to characterize a wide variety of tissues and their microstructure [11], [14]–[17], [22]–[23]. Linear regression analysis of a spectrum can provide information on parameters such as mid-band fit, slope, and 0-MHz intercept values, which correspond to backscatter intensity, the size of acoustic scatterers, and the concentration of acoustic scatterers, respectively [11], [14]–[17], [22]–[24]. These parameters can be used to identify specific tissue characteristics or detect microscopic and macroscopic changes in tissues over time. This method has been applied to various tissue types such as liver, bladder, breast, and prostate tumors, which have all demonstrated unique quantitative ultrasound parameters using both high and low-frequency ultrasound [11], [23]–[25].

The study here is an addition to previous studies on quantitative ultrasound of cell death, and examines the ability to acoustically detect cell death caused by tumor exposure to ultrasound-stimulated microbubble treatments combined with radiation, and examines the extent of the sensitivity of this method. This study supports previous findings which have demonstrated that ultrasound-stimulated microbubbles trigger endothelial cell apoptosis within tumor microvasculature and facilitate radiation enhancement [26]–[28]. Here, the sensitivity of the quantitative method was tested by varying treatment parameters known to influence cell death, such as increasing the concentration of microbubbles, ultrasound pressure, and radiation dose [27]. Results from 25 MHz ultrasound spectral analysis of *in vivo* tumours are compared to histological sections of excised tumor samples. Spectral changes determined within a −6 dB acoustic window about the center frequency were identified to be closely associated with cell death histological changes for various treatment conditions. The results of this study demonstrated significant correlations between these ultrasound parameters and cell death resulting from vascular disruption caused by the microbubble-stimulated ultrasound and radiation treatments. Furthermore, they suggest that quantitative ultrasound imaging can be used as a sensitive tool for characterizing cell death, and is able to detect cellular changes caused by low doses of radiation enhancing agents.

## Methods and Materials

All animal experiments presented in this manuscript were conducted in compliance with Canadian Council on Animal Care guidelines. Experimental protocols were approved by the Committee on the Ethics of Animal Experiments of Sunnybrook Health Sciences Centre. The title of the Animal Use Protocol (AUP #: 13-395 AUP Expiry: 14 April 2014) was as follows: “Novel Ultrasound Microbubble Potentiated Radiosensitization of Tumours.”

### Cell System and Animal Preparation

Tumor-bearing animals were prepared and handled as previously described [26]–[27]. In summary, five male SCID mice bearing PC-3 xenografts were used per condition and a total of 30 different treatment conditions were investigated (described further below). For experiments mice were anesthetized by oxygen ventilated isoflurane and intraperitoneal injection of anesthesia (ketamine and xylazine) as described before [19] prior to imaging and treatment. Animals were sacrificed 24 hours after treatment using euthanyl (0.4 mg/kg).

### Microbubble Preparation and Ultrasound Treatment

Ultrasound-stimulated microbubble treatments were conducted as previously described in [26]–[27]. In short, Definity Perflutren lipid microspheres (Lantheus Medical Imaging, N. Billerica MA, USA), at a concentration of 8 µL/kg (0.2 µL), 80 µL/kg (2 µL), or 1000 µL/kg (25 µL) (or 0.01%, 0.1%, and 1.0% v/v respectively), were used (Clinically recommended imaging dose Definity is 10 µL/kg). An ultrasound treatment system with a planar 500 kHz immersion transducer was used to insonify specimens in order to stimulate microbubbles in the tumor vasculature. The tumor-bearing leg of the mouse was submerged in 37°C water and positioned at a distance of 8.5 cm from the transducer, where the maximum focused acoustic signal (beam width of 31 mm) was achieved. Microbubbles were administered through tail-vein-catheter followed by a saline flush, and the tumors were immediately exposed to ultrasound for five minutes, as was described previously [26]–[27]. For the total treatment time of five minutes the ultrasound exposure time was 750 ms (150 periods×5 ms), resulting in an average duty cycle of 0.25%. Peak negative pressures of 250 kPa, 570 kPa, and 750 kPa were applied using a calibrated ultrasound transducer.

### Radiation Treatment

Radiation treatments were performed as previously described [26]–[27]. In short, following ultrasound treatment, tumors were immediately irradiated using an irradiation cabinet (Faxitron, Wheeling Illinois, USA). X-Rays were delivered at doses of 0 Gy, 2 Gy, or 8 Gy at a dose rate of 200 cGy/minute and an energy of 160 kVp. Mice were shielded with a 3 mm thick lead sheet during irradiation, so that only the tumor region was irradiated.

### Ultrasound Imaging with High Frequency Ultrasound

Conventional high frequency ultrasound imaging was performed prior to and 24 hours after treatment. For each scan, radiofrequency data and B-mode images were obtained volumetrically for tumor specimens *in vivo*. Image acquisition was performed using a Vevo770, (VisualSonics Inc., Toronto, ON, Canada) fitted with a RMV (Real-time Micro Visualization) scanhead, 25 MHz single-element transducer (62 µm resolution) (VisualSonics Inc., Toronto, ON, Canada). Tumors were imaged in the hip-to-toe direction with the transducer positioned approximately 2 mm above skin surface. An average of 60 frames was acquired, per scan, with 250 lines of radiofrequency per frame and a 0.2 mm scan plane separation. For analyses ROI sizes were rectangular shaped and equivalent from sample to sample. These occupied 2/3 of the tumour diameter in width and height.

Spectral parametric maps were generated using in-house software. Mid-band fit values were mapped within a selected region of interest that outlines the tumor. Regions of interest were selected in a consistent manner, which covered the whole tumor, excluding skin and muscle. The analysis bandwidth ranged from 18 to 30 MHz (−6 dB bandwidth). A sliding window analysis rooted in a Hamming function was used [11]. For these images only, ROI's were shaped to the interior of the tumour to show the heterogeneity within tumour samples.

### Histology Preparation

Tumor tissue was prepared as described in [27]. After excision, tissue samples were fixed, processed and embedded in paraffin. Separate tumor sections were stained with hematoxylin and eosin (H&E), or labeled with *in situ* end labelling (ISEL), or cyclophilin A. Unfixed tissue samples were embedded in OCT medium and immersed in liquid nitrogen, and were then stored at −80°C and frozen sections were prepared for ceramide labelling. The immuno-staining procedure for ceramide and cyclophilin A was performed according to the immunohistochemical staining protocol of frozen sections from Relia Tech (Wolfenbüttel, Lower Saxony, Germany). For staining a 10× diluted mouse monoclonal primary antibody for ceramide (Alx-804-196-T050, Enzo Life Sciences, CA-Brockville, ON, Canada) and 100× diluted polyclonal primary antibody (mouse, rat, human) for cyclophilin A (ab41684, Abcam, Toronto, ON, Canada) were used with Histostain-Plus Kit (AEC, Broad spectrum, Cat No. 85-9943) (Life Technologies Inc., Burlington, ON, Canada).

### Spectral Analysis of Radiofrequency Data

Radiofrequency data were analyzed using normalized power spectra as previously described [11]. Normalization was achieved using a reference power spectrum that was acquired from a flat quartz plate. Power spectra were calculated as an average within a selected region of interest. For each spectrum, a linear regression was performed within the −6 dB wide spectral window centered about a center frequency of 25 MHz. From the line of best fit, three quantitative measures, mid-band fit, slope, and 0-MHz intercept were determined and used to characterize responses of samples to treatment.

### Quantifying Relative Fraction of Structurally Altered Nuclei

Cell death was assessed using low-magnification images of ISEL stained tumors captured under a light microscope attached to a CCD camera as previously described [27]. Quantification of cell death-disruption was obtained as described previously [27]. Higher magnification images of H&E stained tumors were obtained using a Leica DM LM (Leica, Concord, Ontario) microscope to detect cellular morphology. Quantification of response was assessed by manually counting the number of structurally altered nuclei, and condensed apoptotic bodies, with a tally counter. Five regions of interest were selected per section and the condensed or fragmented nuclei were counted and normalized to the control to generate relative response values.

### Statistical Analysis

A 2-way ANOVA (Two-factor without replication, α = 0.05) was performed in order to test for statistical significance in each spectral parameter. The same was applied for changes in relative fraction of structurally altered nuclei representing an index of histologically detected cell death. GraphPad Prism Software Version 4 (GraphPad Software, La Jolla, CA, USA) was used.

## Results

In general, quantification of ultrasound data enabled the detection of changes in tumors generated by a range of ultrasound-stimulated microbubble treatment exposures with varying ultrasound intensities and radiation doses. This resulted in closely corresponding ranges of cell death. Statistical tests of significance referenced below are presented in Tables 1–5.

**Table 1:** Results of statistical analysis performed on the “*Average Changes in Midband-fit, 0-MHz Intercept, and Slope parameter*” using 2-way ANOVA without replication.

	*2-WAY ANOVA*		
	Fixed Parameter	Source of Variation	*P*-value
**MBF**	P = 250 kPa	Radiation	<0.0001
		[MB]	<0.0001
	P = 570 kPa	Radiation	<0.0001
		[MB]	<0.0001
	P = 750 kPa	Radiation	<0.0001
		[MB]	<0.0001
**0-MHz Intercept**	P = 250 kPa	Radiation	<0.0001
		[MB]	<0.0001
	P = 570 kPa	Radiation	<0.0001
		[MB]	<0.0001
	P = 750 kPa	Radiation	<0.0001
		[MB]	<0.0001
**Slope**	P = 250 kPa	Radiation	<0.0001
		[MB]	<0.0001
	P = 570 kPa	Radiation	<0.0001
		[MB]	<0.0001
	P = 750 kPa	Radiation	<0.0001
		[MB]	<0.0001

**Table 2:** Results of statistical analysis performed on the “Quantification of Cell Death” using 2-way ANOVA without replication.

*2-WAY ANOVA*		
Fixed Parameter	Source of Variation	*P*-value
P = 750 kPa	Radiation	<0.05
	[MB]	<0.0001

**Table 3:** Results of statistical analysis performed on the “Quantification of normalized fraction of condensed or fragmented nuclei” using 2-way ANOVA without replication.

*2-WAY ANOVA*		
Fixed Parameter	Source of Variation	*P*-value
P = 250 kPa	Radiation	<0.0001
	[MB]	<0.0001
P = 570 kPa	Radiation	<0.0001
	[MB]	<0.0001
P = 750 kPa	Radiation	<0.0001
	[MB]	<0.0001

**Table 4:** Results of statistical analysis performed on the “*Quantification of normalized fraction of condensed or fragmented nuclei*” using one-tailed paired t-test.

Paired t-test (one tailed)					
At 250 kPa (for 0 Gy, 2 Gy, 8 Gy)		At 570 kPa (for 0 Gy, 2 Gy, 8 Gy)		At 750 kPa (for 0 Gy, 2 Gy, 8 Gy)	
Comparison	*P*-value	Comparison	*P*-value	Comparison	*P*- value
0 vs 8 µL/kg	<0.05	0 vs 8 µL/kg	<0.05	0 vs 8 µL/kg	<0.05
8 vs 80 µL/k	<0.05	8 vs 80 µL/kg	<0.05	8 vs 80 µL/kg	<0.05
80 vs 1000 µL/kg	<0.05	80 vs 1000 µL/kg	<0.05	80 vs 1000 µL/kg	<0.05

**Table 5:** Results of statistical analysis performed on the “Quantification of Ceramide” using 2-way ANOVA without replication.

*2-WAY ANOVA*		
Fixed Parameter	Source of Variation	*P*-value
P = 750 kPa	Radiation	<0.05
	[MB]	<0.05

### Increases in Radiation Dose, Microbubble Dose, or Rarefactional Ultrasound Pressure Cause Increases in Ultrasound Backscatter Intensity from Tumor

**Figure 1:**
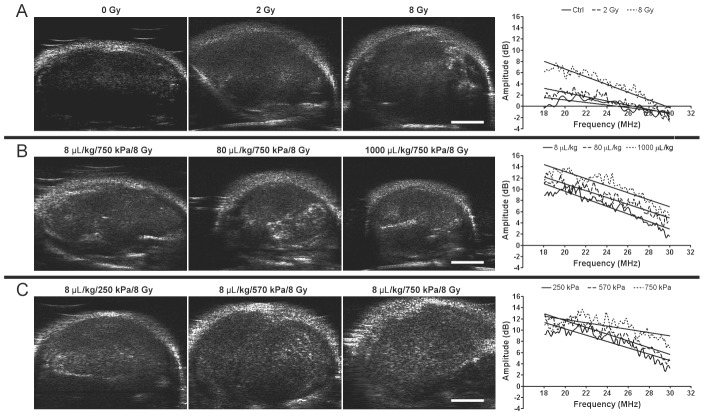
High-frequency ultrasound B-mode images of PC-3 xenografts. B-Mode (left) and on the right side of each row, respective representative normalized power spectra are shown. (A) Tumors treated with varying radiation doses (0–8 Gy) without ultrasound-microbubble treatment. (B) Tumors treated with varying microbubble concentrations (8–1000 µL/kg) combined with 750 kPa of ultrasound pulse and 8 Gy-radiation. (C) Tumors treated with varying ultrasound pressures (250–750 kPa) combined with 8 µL/kg of microbubbles and 8 Gy-radiation. The scale bar represents 2 mm.

Results demonstrated that higher radiation doses caused increased cell death, resulting in higher backscatter intensity. Figure 1A displays representative B-mode images and corresponding power spectra of untreated tumors and tumors treated with either 2 Gy or 8 Gy of radiation. In the B-mode images increases in intensity, indicative of higher backscatter, were visible within tumors receiving higher radiation doses. This was verified quantitatively in tumors treated with higher radiation doses which demonstrated spectra with greater overall signal amplitudes (Figure 1A). Tumors irradiated with 2 Gy and 8 Gy showed approximately 0.9±2.0 dB and 3.7±1.8 dB increases in mid-band fit values compared to the control, respectively.

Tumors treated with higher dose of ultrasound-stimulated microbubbles similarly demonstrated greater backscatter intensity (Figure 1B and C). Mid-band fit values indicated 3.4±2.3 dB and 7.0±3.6 dB increases for 8 µL/kg , for 80 µL/kg and 1000 µL/kg microbubble concentrations, respectively (Figure 1B). Likewise, increases in rarefactional ultrasound pressure for microbubble stimulation resulted in specimens *in vivo* with elevated backscatter intensity. The increases ranged from 1.4±3.3 dB to 3.4±5.0 dB when comparing 250 kPa to 570 kPa and 750 kPa, respectively (Figure 1C). A “plateau” of the backscatter intensity was observed at a pressure of 570 kPa and higher.

### Spectral Parameters of Ultrasound Signal Show Sensitivity to Changes in Radiation Dose, Microbubble Concentration, and Rarefactional Ultrasound Pressure

**Figure 2:**
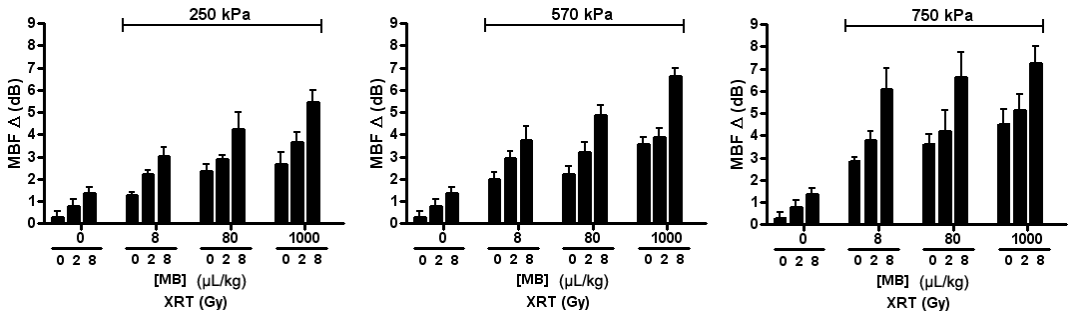
Average changes in midband-fit parameter. Each bar represents the mean of midband-fit values of five mouse-borne tumors (n = 5). The error bar indicates the standard error within the sample size. Statistical testing using 2-way ANOVA indicates the effects caused by the changes in both microbubble concentration and dose of radiation to be very significant for every graph (*p*<0.0001). Each graph shows the average changes in midband-fit for varied microbubble concentration and radiation doses at a fixed ultrasound pressure: 250 kPa, 570 kPa, and 750 kPa.

**Figure 3:**
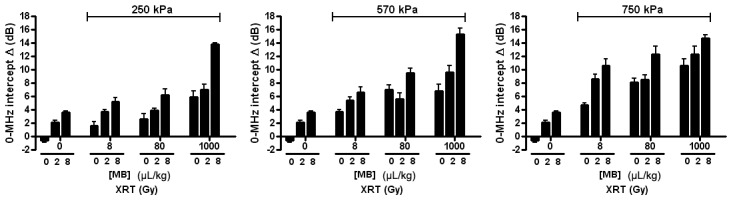
Average changes in 0-MHz intercept parameter. Each bar represents the mean of 0-MHz intercept values of five mouse-borne tumors (n = 5). The error bar indicates the standard error within the sample size. Statistical testing using 2-way ANOVA indicates the effects caused by the changes in both microbubble concentration and dose of radiation to be very significant for every graph (*p*<0.0001). Each graph shows the average changes in 0-MHz intercept for varied microbubble concentration and radiation doses at a fixed ultrasound pressure: 250 kPa, 570 kPa, and 750 kPa.

**Figure 4:**
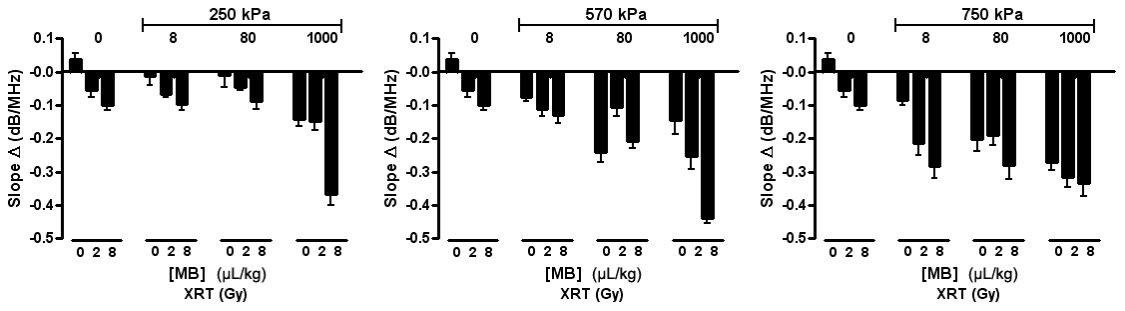
Average changes in slope parameter. Each bar represents the mean of slope values of five mouse-borne tumors (n = 5). The error bar indicates the standard error within the sample size. A statistical test using 2-way ANOVA indicates the effects caused by the changes in both microbubble concentration and dose of radiation to be very significant for every graph (*p*<0.0001). Each graph shows the average changes in slope for varied microbubble concentration and radiation doses at a fixed ultrasound pressure: 250 kPa, 570 kPa, and 750 kPa.

Results demonstrated that mid-band fit, slope, and 0-MHz intercept spectral parameters were strongly correlated with increases in radiation dose, microbubble concentration, and ultrasound pressure (*p*<0.0001). Overall, the average change in mid-band fit increased as radiation dose, microbubble concentration, or microbubble-stimulating ultrasound responses were increased (Figure 2). The trend for every parameter was nearly linear for the lowest ultrasound pressure, 250 kPa, whereas with the highest pressure of 750 kPa, results appeared to plateau, especially where the highest change in mid-band fit values was 7.0±4.1 dB, resulting from the combined treatment of 1000 µL/kg of microbubbles stimulated at 750 kPa and a radiation dose of 8 Gy. However, the treatment condition using the near clinically-utilized (for imaging) microbubble concentration (8 µL/kg of microbubbles, combined with 750 kPa, 2 Gy radiation) resulted in a 3.5±2.4 dB increase in mid-band fit. The average change in 0-MHz intercept, linked to the concentration of scatterers, demonstrated trends analogous to the mid-band fit (Figure 3). The 0-Mhz parameter displayed increases when radiation dose, microbubble concentration, or ultrasound pressure were increased. In conditions where the highest microbubble concentration was administered, the 0-MHz intercept spectral parameter was high regardless of the applied ultrasound pressure. The “highest-dose” treatment condition had an average change in 0-MHz intercept of 15.4±2.5 dB, while the treatment condition using the lowest microbubble concentration increased by 9.2±3.4 dB, relative to control. However, the average change in slope, which can be linked to the size of the scatterers appeared inversely correlated with increasing treatment dose (Figure 4). It also exhibited an apparent plateau when increasing microbubble dose under the highest ultrasound pressure.

**Figure 5:**
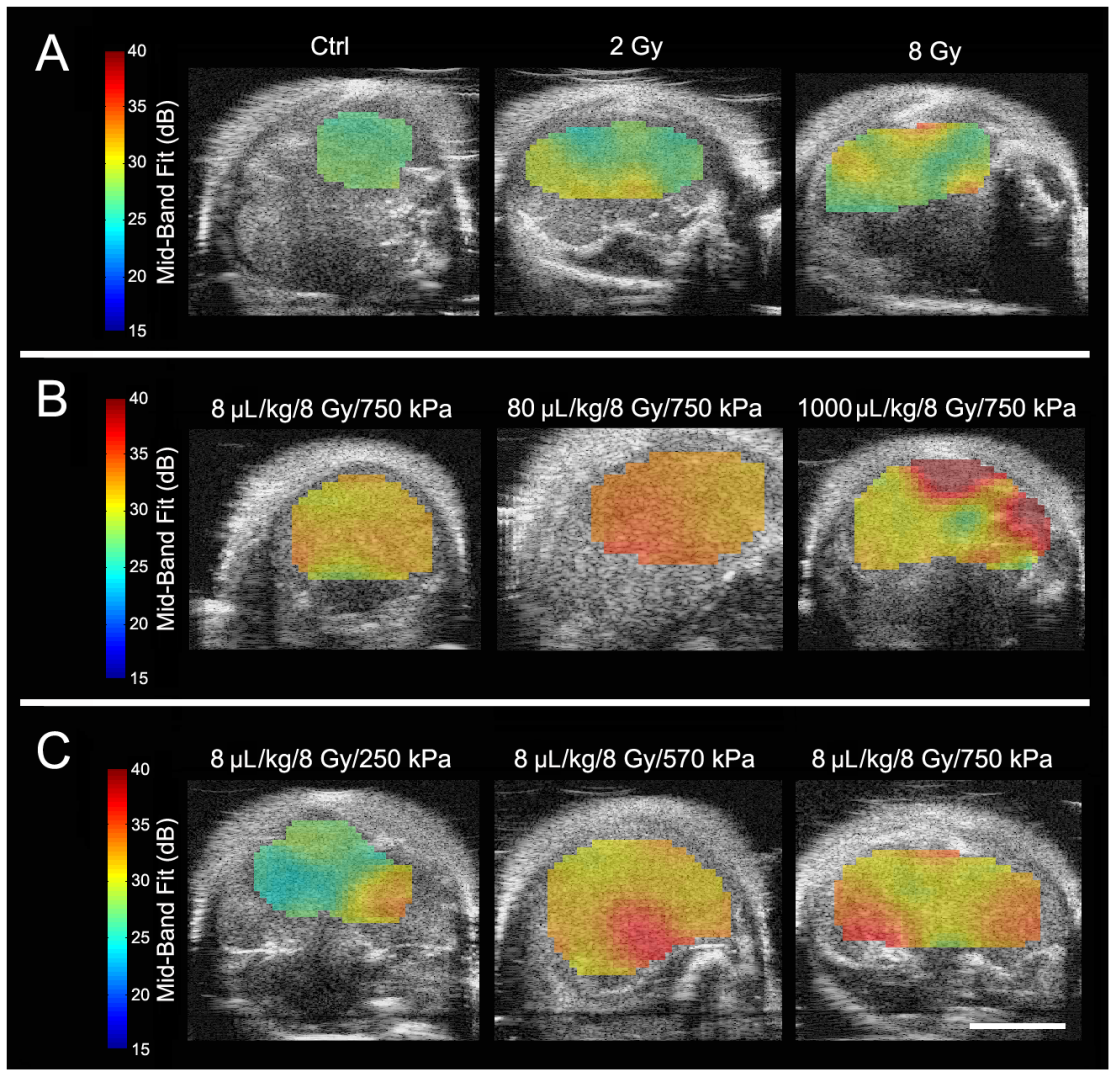
High frequency ultrasound B-mode images with ROI parametric overlays of the mid-band fit biomarker for PC-3 xenografts. (A) Tumors treated with varying radiation doses (0–8 Gy) without ultrasound-microbubble treatment. (B) Tumors treated with varying microbubble concentrations (8–1000 µL/kg) combined with 750 kPa of ultrasound pulse and 8 Gy-radiation. (C) Tumors treated with varying ultrasound pressures (250–750 kPa) combined with 8 µL/kg of microbubbles and 8 Gy-radiation. The scale bar represents 4 mm.

Parametric maps of mid-band fit demonstrated variations of ultrasound backscatter on a spatial basis (Figure 5). Within each tumor they provided a means of discriminating treatment response. The results indicated graphically that the higher the radiation dose, ultrasound pressure, or microbubble concentration, the higher the mid-band fit was. Whereas the untreated tumor exhibited mid-band fit values ranging from 28.5 dB to 30 dB, the tumor that received the highest radiation (8 Gy) exhibited those from 28.5 dB to 35.5 dB. The increase in ultrasound pressure from 250 kPa to 750 kPa under fixed microbubble concentration and radiation dose exhibited an increased power spectrum window from 28.5–35 dB to 32.5–35 dB. Likewise, the increase in microbubble concentration from 8 µL/kg to 1000 µL/kg, under fixed radiation dose and ultrasound pressure, demonstrated an increase from 31.5–36.2 dB to 36.0–37.5 dB. However, responses were not uniform throughout the tumor and increased with heterogeneous “hot spots” of tumors. This is linked to the histological observations below.

### Immunohistochemical Characterization Parallel Spectral Analyses: Increases in Microbubble and Radiation Dose Lead to Enhanced Cell Death

**Figure 6:**
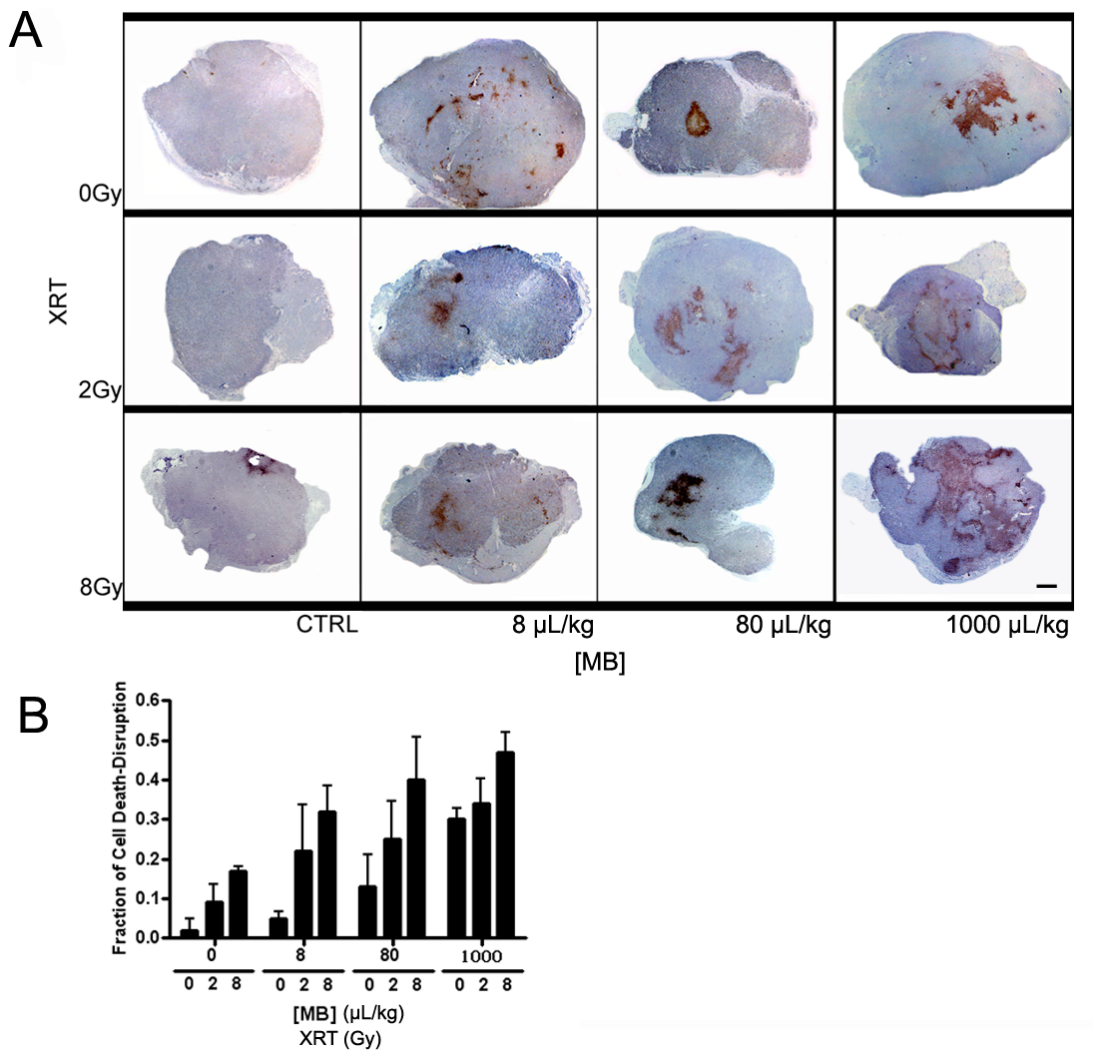
Low magnification light microscope images of ISEL-labeled PC-3 xenografts. (A) In addition to control, tumors treated with ultrasound pulses at 750 kPa and varied radiation doses and microbubble concentrations are shown. For each panel L, M, and H represent low (8 µL/kg), medium (80 µL/kg), and high (1000 µL/kg) microbubble concentrations, respectively. The panel shows tumors with ISEL staining that exemplify non-affected cells as bluish purple and DNA damaged cells as reddish brown. The scale bar represents 1 mm. (B) Quantification of fraction of cell death and disruption. The graph demonstrates the fraction of cell death-disruption for various microbubble concentration and radiation doses at a fixed ultrasound pressure: 750 kPa. For each treatment condition the average value of the fraction of cell death-disruption of five mice (n = 5) has been plotted with an error bar indicating the standard error within the sample size. Statistical testing using 2-way ANOVA indicates effects caused by the changes in both microbubble concentration and dose of radiation to be significant (*p*<0.05).

Low magnification images of ISEL labelling demonstrated that increases in microbubble concentration and radiation dose results in increased cell death in tumors (Figure 6A). At the highest radiation dose and ultrasound pressure, the fraction of regions of damaged DNA increased from approximately 0.1 to 0.4 as microbubble dose increased from low (8 µL/kg) to high (1000 µL/kg) (Figure 6A). Quantitative analysis demonstrated an average increase in cell death-disruption (n = 5) from 34±7% to 47±5% for the identical treatment (Figure 6B). Analysis by 2-way ANOVA tests verified that the trends shown in increasing microbubble concentration and dose of radiation at fixed ultrasound pressures were statistically significant by (*p*<0.05). At the highest microbubble concentration and ultrasound pressure, cell death increased from 0.2 to 0.4 from 0 Gy to 8 Gy radiation, respectively. Responses appeared sparsely heterogeneous throughout tumor tissue with central core of tumors exhibiting the most response.

### Quantification of Cell Death Demonstrates Increases as Microbubble Concentration, Rarefactional Ultrasound Pressure, or Radiation Dose Increase

**Figure 7:**
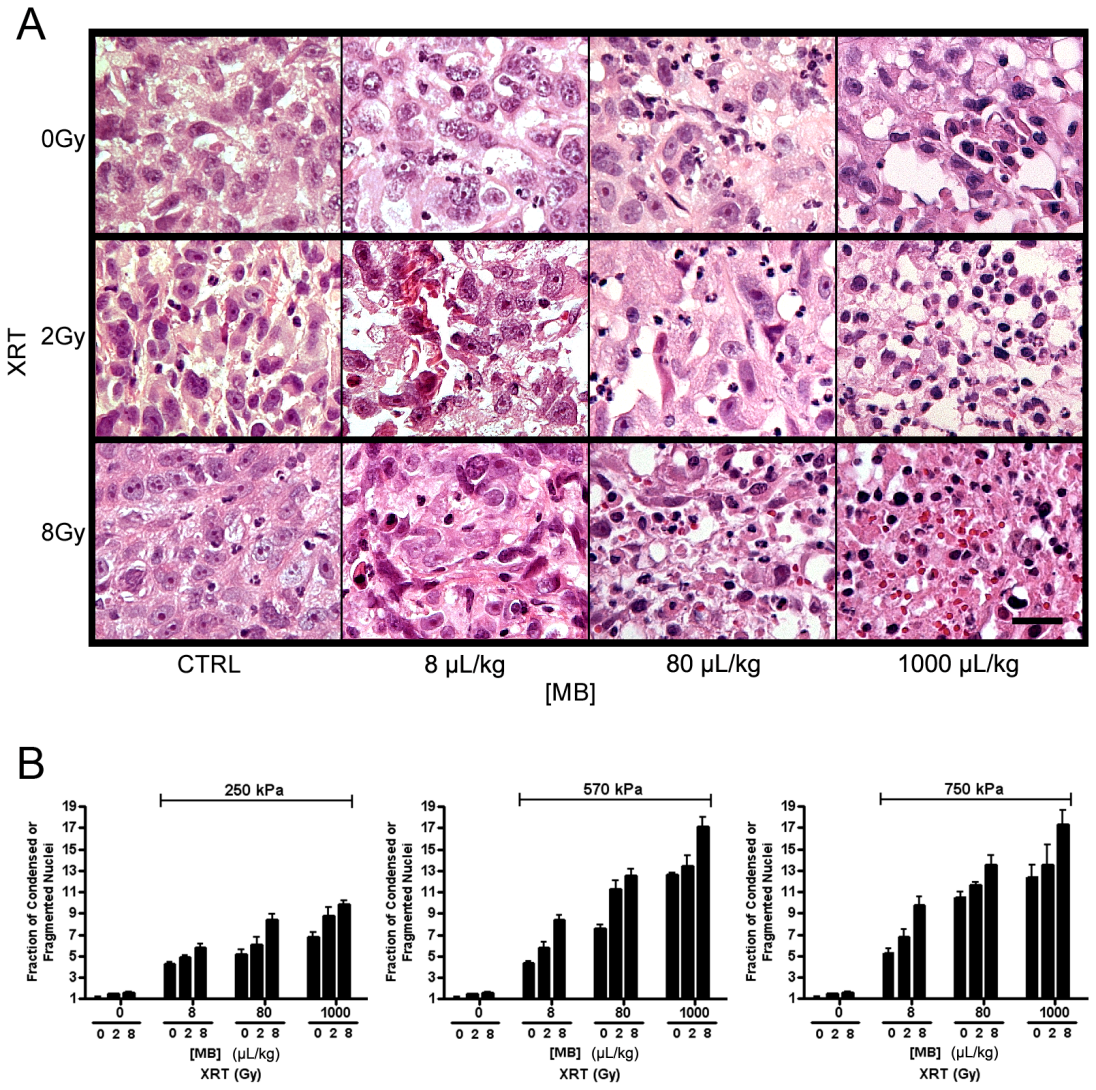
High magnification light microscope images of H&E-stained PC-3 xenografts. In addition to control, tumors treated with ultrasound pulses at 750(A) The panel shows H&E stained cells that exhibit condensed and/or fragmented apoptotic bodies. Each Panel demonstrates a representative region of cell death within tumor. Microbubble concentrations are given as 8 µL/kg, 80 µL/kg, and high 1000 µL/kg. The scale bar represents 25 µm. (B) Quantification of normalized fraction of condensed or fragmented nuclei. Each graph shows the fraction of cell death and disruption for varied microbubble concentration and radiation doses at a fixed ultrasound peak-negative pressure: 250 kPa, 570 kPa, and 750 kPa. Each bar represents the mean value of five samples (n = 5) and the error bar indicates the standard error. Statistical testing using 2-way ANOVA indicates the effects caused by the changes in both microbubble concentration and dose of radiation to be very significant (*p*<0.0001).

High magnification images of H&E stained sections from treated and control tumors were acquired in order to examine areas of cell death (Figure 7A). The purpose was to observe the morphological changes within cells and consider potentially related biological changes in the ultrasound signal in the context of previous work. Normal cells appeared with intact nuclei whereas cells affected by therapy appeared with condensed or fragmented nuclei (Figure 7A) consistent with cell death. Results indicated that the fraction of fragmented nuclei resembling (in part) apoptotic bodies increased with higher microbubble concentration or radiation dose. Fragmented and condensed nuclei were quantified in order to verify whether the concentration of potential scatterers derived from spectral analysis were correlated to number of putative histologically-detected apoptotic bodies (Figure 7B). Results indicated increasing proportions of apoptotic bodies with higher microbubble concentration, increasing radiation dose, or greater ultrasound pressure. The most disruptive treatment condition (1000 ul/kg of microbubbles, 750 kPa, 8 Gy of radiation) resulted in a 17-fold increase in apoptotic bodies and destroyed cells, relative to the control. Lower exposure treatment conditions (e.g. 8 µL/kg of microbubbles, 750 kPa, 2 Gy radiation) resulted, in contrast, in a 7-fold increase in apoptotic bodies.

### Ceramide-Mediated Apoptosis is a Primary Biochemical Mechanism Behind Radiation Treatment Combined with Ultrasound-Stimulated Microbubbles

**Figure 8:**
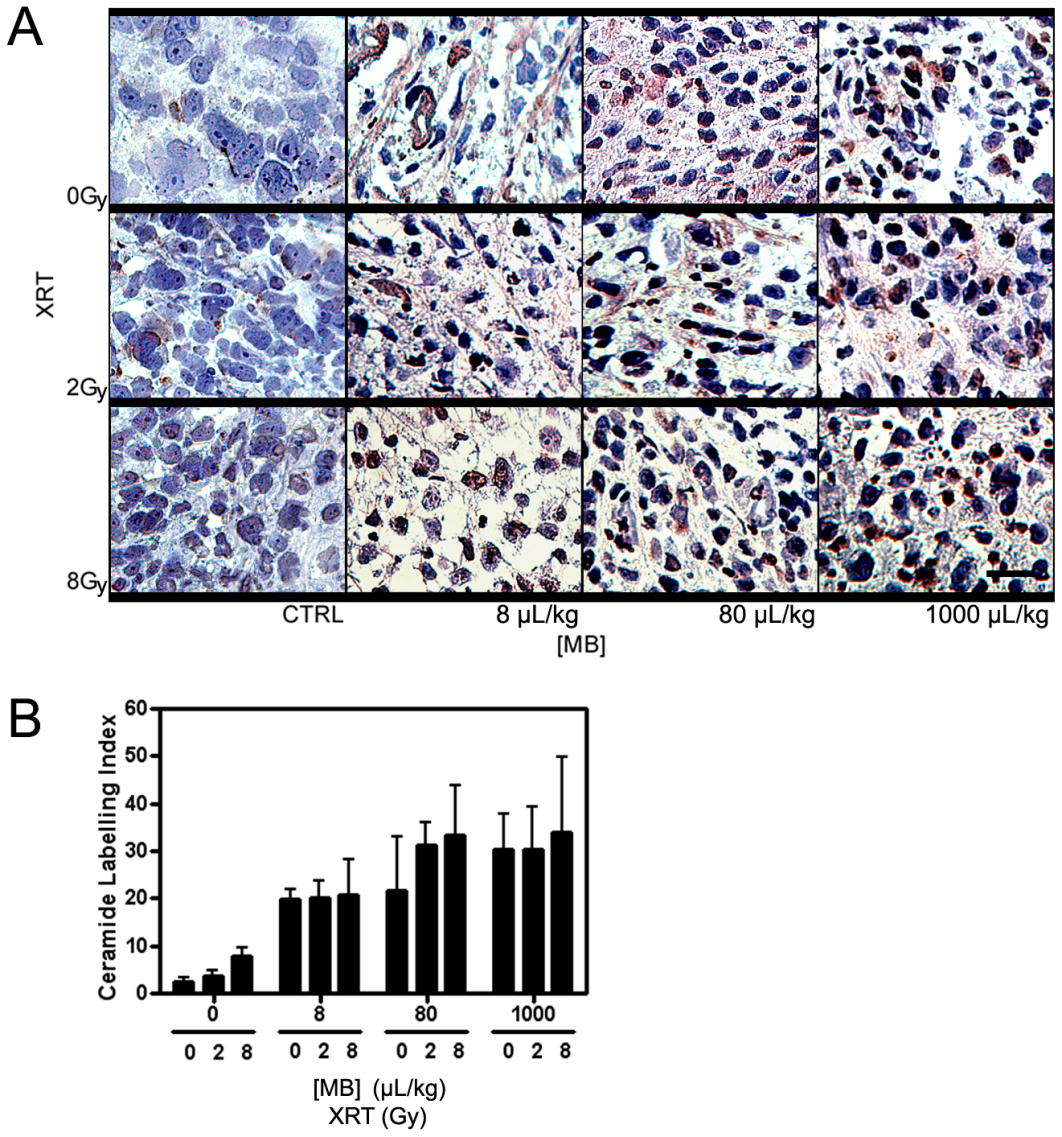
High magnification light microscope images of PC-3 xenografts immuno-stained with ceramide. (A) Tumors treated with ultrasound at 750 kPa and varied radiation doses and microbubble concentrations are shown. Ceramide is stained as brown that it can be distinguished from the bluish purple counter-staining. Each panel demonstrates the most representative region of cell death occurred in the tumor. Microbubble concentrations are given as 8 µL/kg, 80 µL/kg, and 1000 µL/kg. The scale bar represents 25 µm. (B) Quantification of ceramide production is shown. The graph shows the ceramide labelling index for various microbubble concentrations and radiation doses at a fixed ultrasound pressure of 750 kPa. Each bar represents the mean value of five regions of interest and the error bar indicates the respective standard deviation. 2-Way ANOVA indicates results related to the increase in microbubble concentration to be significant (*p*<0.05).

Previous studies have demonstrated that ceramide primarily mediates this ultrasound-stimulated apoptotic cell death in endothelial cells [26]–[27]. Ceramide is a cell-stress marker that is typically observable after treatments with single high radiation doses (>6 Gy) or treatments equivalent to the stress effects of such radiation doses. Results demonstrated increasing amounts of ceramide within cells as the dose of radiation, microbubble concentration, or ultrasound pressure were increased (Figure 8). At 750 kPa of ultrasound pressure, the extent and intensity of ceramide within cells increased as radiation dose and microbubble concentration were increased (Figure 8A). Quantification of ceramide-staining by immunohistochemistry demonstrated that an ultrasound-stimulated microbubble treatment without radiation or with a low radiation exposure can produce ceramide but less than that for high radiation exposures (>6 Gy) (Figure 8B).

### Permeabilization of Cells is Enhanced Post Ultrasound-Stimulated Microbubble Treatment Combined with Radiation

**Figure 9:**
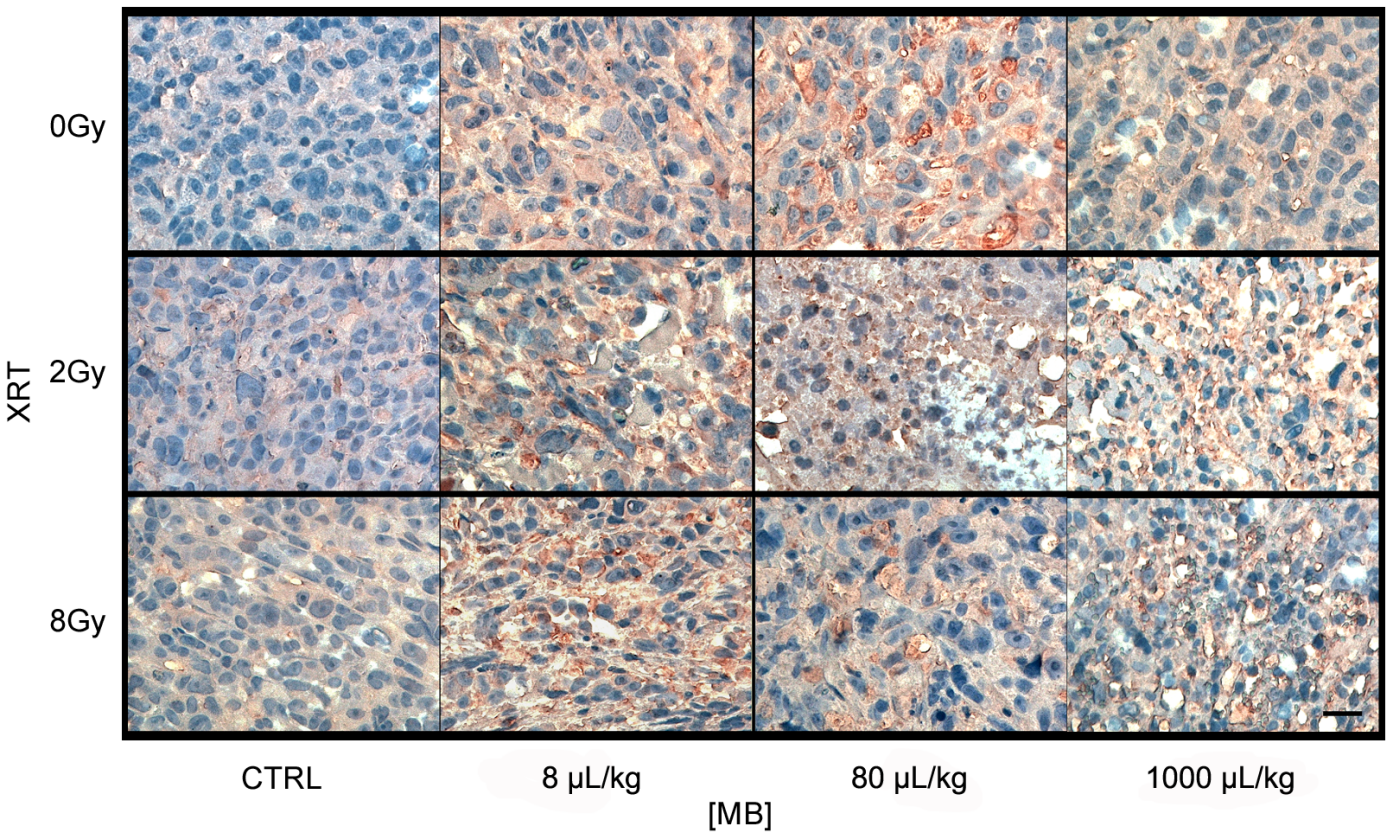
High magnification light microscope images of PC-3 xenografts immuno-stained with cyclophilin A. Tumors treated with ultrasound pulses at 750-staining. Each panel demonstrates a representative region of cell death occurred in the tumor. Microbubble concentrations are given as 8 µL/kg, 80 µL/kg, and 1000 µL/kg. The scale bar represents 25 µm.

Previous literature has indicated a proportional relation between the permeabilization of cells and ultrasound treatment. The higher the pressure or the lower the centre frequency of ultrasound, the more cell permeabilization can be achieved [29]–[30]. In addition, the effects of increasing microbubble concentration or radiation dose on cell-permeabilization have been assessed in this study (Figure 9). Cyclophilin-A labelling, which is used to mark vascular remodeling or endothelial cell membrane permeabilization [31], was performed and results imaged with high magnification light microscopy. In the presence of the high ultrasound pressure, 750 kPa, most of the endothelial cells appeared to be permeabilized regardless of microbubble concentration and radiation dose. Tumors not receiving ultrasound-stimulated microbubble treatment exhibited minimal cellular permeabilization. Cells that were only irradiated also generated more cyclophilin A in comparison to control.

## Discussion

This investigation supports the finding that ultrasound-based spectral parameters, such as the MBF parameter and 0-MHz intercept, can detect changes associated with cell death consistent with previously described research [11], [14]–[17]. It validates the detection sensitivity of these methods when used to reveal changes resulting from varying treatment parameters. It also sets a milestone for developing quantitative ultrasound imaging into a potential non-invasive cell monitoring tool for novel radiation enhancing anti-vascular therapies. The biological system used here is one of cell disruption caused by variations of ultrasound-stimulated microbubble treatment, combined with radiation treatment [27]. This study explores the correlation between ultrasound parameters and cell-death extent, which can be ultimately used to develop ultrasound imaging into a non-invasive surrogate to biopsy, to assess cell-death related tumor responses. The results here provide an important groundwork for creating dual-purpose ultrasound-based therapeutic and imaging, combining radiosensitization-capabilities and cellular-structure sensitive functional quantitative imaging to non-invasively monitor the treatment effects. The ultrasound-based “biomarkers” generated from acoustic spectra (MBF, 0-MHz intercept, and slope) exhibited adequate sensitivity to detect cellular changes induced by these ultrasound and microbubble treatment conditions.

In other studies, prostate cancer cells treated with chemotherapeutic agents have been subjected to ultrasound imaging, both *in vitro* and *in vivo*, and assessed for changes in intracellular structures [11], [15]–[16]. Chemotherapy is recognized to cause various distinctive morphological changes within cells that are characteristic of disruptions in biochemical pathways [32]–[34]. Cell death features such as enlargement of cells with a vacuolated cytoplasm and angular hyperchromatic nuclei [34], or other features such as fragmented nuclei indicative of cell death due to apoptosis were observed in this study as well as others [21]. Other studies treating tumors with photodynamic therapy, radiation therapy, and chemotherapy in multiple tumor types indicated similar ultrasound analyses that were linked to cellular morphology changes [17]. Investigations have demonstrated that the condensation of nuclei is a structural change that is a major potential determinant of increased ultrasound backscatter with treatment [11], [14]–[17]. This is posited because nuclear condensation results in the greatest potential changes to the elasticity and density of the nucleus in the cell.

Early permeabilization of plasma membrane was identified here using cyclophilin A immuno-stain (Figure 9) [31]. The results demonstrated that in the presence of the highest ultrasound pressure, 750 kPa, and ultrasound-stimulated microbubbles, all of the tumors were relatively homogeneously permeabilized regardless of microbubble concentration and radiation dose. Permeabilization increased as these parameters were increased to their maximum in this study, however changes did not appear to be linearly proportional to the microbubble concentration or radiation dose. The tumors that were not treated or treated with radiation only also demonstrated, similarly, increased amounts of cyclophilin A following an ultrasound-stimulated microbubble treatment or an enhanced radiation treatment, respectively. This suggests that the trend in cell death shown here was potentially independent from the effect of permeabilization.

In the study here, treatment with ultrasound-stimulated microbubbles combined with radiation was used as the primary treatment and has been identified to cause rapid cell death, in terms of structural changes within cells (24 hours). Here, destruction of tumor vasculature leading to secondary tumor cell death was the mechanism for cellular changes. In these treatments, low doses (<2 Gy) of radiation combined with ultrasound-stimulated microbubbles have exhibited comparable efficiency as high doses of radiation only (>5 Gy), linked to ceramide-mediated apoptosis in endothelial cells within tumor vasculature [26]. As expected, more intense treatments (higher radiation dose, microbubble concentration, and ultrasound pressure) in this study resulted in greater amounts of cell death leading to ultrasound backscatter changes. For all treatment conditions, increases in average changes in spectral parameters such as mid-band fit and 0-MHz intercept were observed. These corresponded with increases in average backscatter intensity and were linked to increases in dead appearing or apoptotic appearing cells, respectively. The trends were in agreement with the histological results here, as assessed in more depth previously [27]. The use of the pressure of 750 kPa caused rapid increases in cell death when combined with increasing microbubble concentration, which eventually appeared to plateau. As previously suggested, this implies a potential saturation effect due to pressure level (>570 kPa) leading to microbubble collapse [27]. The size of ultrasound scatterers has been previously reported to be correlated with changes in spectral slope [23]–[24]. Changes in spectral slope were also observed here, where higher treatment doses resulted in a more negative slope (Figure 4). Although the condensed and fragmented structures derived from apoptosis are generally much smaller in size than nuclei from normal intact cells and in homogenously responding samples slope may increase, we have demonstrated previously that in heterogeneous samples with patches of response, or responses with large amounts of necrosis, similar to that here, slope decreases can occur. In a previous investigation that assessed spectral parameters at different times (0, 4, 12, 24, 48 h) after chemotherapy exposure, changes in the trend in spectral slopes were observed over time [11]. Results demonstrated increasing slope leading up to a 12 hour-point, then a horizontal slope at the 12 hour-point, and a decreasing slope between the 12 hour-point to 48 hour-point. This was correlated with the development of necrosis after apoptosis and mixed modes of cell death. The results are complex and affected not only by the size of individual ultrasound scatterers which cannot be inferred from the data but also their compressibility and density, number density of scatterers, and in the case of ultrasound for these wavelengths, where the wavelength is 3–4 times the size of a cell, the spatial distribution of the scatterers.

The study here does not address the causation of the spectral changes observed and only establishes a co-incidence of spectral changes and the development of cell death. Other research has investigated causal factors in the past and is summarized elsewhere [35]. In summary, that research has demonstrated that a working model links changes in ultrasound backscatter with cell death with evidence from multiple sources of experimental results. In short, that evidence includes (as reviewed in [35]):

- In highly cellular xenograft tumours backscatter signals and spectra are identical to backscatter signals and spectra of centrifuged cell models that mimic the histological packing of xenografts. Such packed cell models have no extracellular matrix and collagen present yet exhibit nearly identical ultrasound scattering profiles.
- Different cell types may be differentiated in part on the basis of their nuclear signals. This further suggests an important role of nuclear structure in backscattered ultrasound.
- In cell experiments where nuclear structure is specifically modified, there are changes in backscattered ultrasound. It has been previously demonstrated that such changes specifically cause the types of backscatter changes observed. When cells are treated with colchicine to arrest them in G2/M of mitosis with condensed nuclear material there are significant increases in backscattered ultrasound. It has been demonstrated that such increases can be reversed by digesting cellular DNA through enzymatically treating such colchicine treated cells with DNAse Additionally, if one similarly subjects viable cells or mouse liver tissue to enzymatic digestions with DNAse, backscatter signals drop to at least half their pretreatment values [23].
- Similarly, other experiments in which cells are treated with sodium butyrate to cause chromatin unfolding exhibit significant decreases in ultrasound backscatter.
- Calculated scatterer sizes from ultrasound backscatter do not work out to be the same as cell sizes but coincide with smaller sizes suggestive again of an important role of the nucleus.
- Isolated nuclei from apoptotic cells in comparison to viable cells exhibit greater backscatter (see [35]).

It is speculated that with the treatments here which induce endothelial cell apoptosis, followed by vascular collapse, and then secondary ischemic cell death, after 12 hour necrosis starts to occur alongside apoptosis leading to swelling of organelles including the nuclei and chromatin [21], and subsequently, cells. Therefore, necrotic cells result in potentially larger sized scatterers in contrast to apoptotic cells. In this study, tumors were imaged 24 hour after treatment, when the process of necrosis or mixed apoptosis and necrosis has already begun. Production of cyclophilin A, which is also indicative of necrosis, supports this point as well. Moreover, with greater treatment exposure more necrosis was expected to occur. Thus, it is likely that the aggregates of necrotic bodies caused by enhanced treatment (millimeter-sized patches of dead cells observed), occurring together with apoptotic bodies resulted in a larger scatterer size and a more negative slope change. The large patches of cell death are also expected to act as scattering structures which can also affect frequency-dependent ultrasound spectral parameters. Other changes occur in the treatments here as well, due to the vascular destruction. Cyclophilin A (present here as evident in immunohistochemistry) has been found to be produced in company with reactive oxygen species induced by vascular remodeling [31], [36]–[38].

In order to demonstrate a biological mechanism behind cell death ceramide production was assessed. Ceramide is a lipid-mediator that is often triggered by a variety of stress stimuli such as heat, reactive oxygen species, and ionizing radiation [39], [40]–[41]. It is linked to apoptosis following a caspase-activating mechanism [39], [40]–[41]. A greater production of ceramide was observed within tumor cells following ultrasound-stimulated microbubble treatment and radiation. This pattern mirrored analyses of the average change in mid-band fit and 0-MHz intercept which showed dependency on microbubble dose and ultrasound pressure. It is suggested that during the ultrasound-stimulated microbubble treatments, the perturbation of blood vessels through micro-streaming and consequent shear stresses on endothelial cells, or non-inertial microbubble cavitation trigger ceramide-stimuli [27], [41]–[43]. The results confirm that ceramide production is associated with ultrasound-stimulated microbubble-induced cell death identified by quantitative ultrasound imaging in this study. It is also expected that the architectural randomness within tumor vasculature may contribute to enhancing backscatter intensity. Previously, changes in tumor with treatments that cause vascular remodeling have also resulted in backscatter changes [44].

## Conclusions

In this study SCID mice with human prostate cancer xenografts were treated with different concentrations of microbubbles (8, 80, or 1000 µL/kg), with bubbles stimulated with various ultrasound pressures (250, 570, or 750 kPa) and tumor tissue exposed to radiation (0, 2, or 8 Gy doses). Each tumor was imaged with ultrasound prior to treatment and 24 h after treatment. Spectral analysis results were in agreement with immunohistochemical analysis which demonstrated that there was an increase in backscatter intensity and quantitative ultrasound parameters co-incident with cell death increases with treatment. The increase in backscatter intensity when compared to control samples ranged from 1.9±1.6 dB for the clinically-recommended dose (8 µL/kg, 250 kPa, 2 Gy) to 7.0±4.1 dB for the extreme treatment condition (1000 µL/kg, 750 kPa, 8 Gy). *In situ* end-labelling (ISEL), ceramide and cyclophilin A labelling was performed to identify regions of cell death due to DNA fragmentation, ceramide mediated apoptosis, and permeabilization of cells, respectively.

This type of ultrasound-stimulated radiation-enhancing treatment would offer a great advantage for patients by saving time since less treatment is needed while using quantitative ultrasound to monitor tumors. The former uses ultrasound to stimulate microbubbles to destruct tumor microvasculature and enhance effects of radiation treatment while the latter uses ultrasound to detect such cell death. This preliminary investigation provides significant groundwork for developing a clinical application that benefits patients by offering both ultrasound-mediated microbubble radiosensitization with non-invasive tissue characterization.
